# Transcriptional regulation and drug resistance in *Mycobacterium tuberculosis*

**DOI:** 10.3389/fcimb.2022.990312

**Published:** 2022-09-02

**Authors:** Paolo Miotto, Rita Sorrentino, Stefano De Giorgi, Roberta Provvedi, Daniela Maria Cirillo, Riccardo Manganelli

**Affiliations:** ^1^ Emerging Bacterial Pathogens Unit, Div. of Immunology, Transplantation and Infectious Diseases IRCCS San Raffaele Scientific Institute, Milano, Italy; ^2^ Department of Molecular Medicine, University of Padova, Padova, Italy; ^3^ Department of Biology, University of Padova, Padova, Italy

**Keywords:** Mycobacteria, drug resistance, transcriptional regulation, tuberculosis, sigma factors, riboswitch

## Abstract

Bacterial drug resistance is one of the major challenges to present and future human health, as the continuous selection of multidrug resistant bacteria poses at serious risk the possibility to treat infectious diseases in the near future. One of the infection at higher risk to become incurable is tuberculosis, due to the few drugs available in the market against *Mycobacterium tuberculosis*. Drug resistance in this species is usually due to point mutations in the drug target or in proteins required to activate prodrugs. However, another interesting and underexplored aspect of bacterial physiology with important impact on drug susceptibility is represented by the changes in transcriptional regulation following drug exposure. The main regulators involved in this phenomenon in *M. tuberculosis* are the sigma factors, and regulators belonging to the WhiB, GntR, XRE, Mar and TetR families. Better understanding the impact of these regulators in survival to drug treatment might contribute to identify new drug targets and/or to design new strategies of intervention.

## Introduction

One of the most fascinating aspects of bacteriology is the extremely fast and efficient responsivity of bacteria to external stimuli, which is translated in a fast and precise variation of their transcriptional profile. This capacity is founded on complex regulatory networks based on sigma factors, transcriptional repressors/activators, two component systems, small RNAs or riboswitches able to reshape bacterial physiology allowing the cells to adapt in real time to any external challenge.

Antibacterial drugs can be considered atypical stressors, both causing a direct interference with cellular physiology and inducing secondary stress due to this interference, as in the case of the oxidative stress induced by bactericidal drugs (Kohanski et al., 2007; Martínez et al., 2020). Consequently, bacteria respond to drug with profound changes in their transcriptional profile that can increase bacterial drug resistance (DR) by target overexpression, drug modification, induction of efflux systems, or simply by helping the cells to respond to the drug-induced stress.

To be noted that whereas DR is usually associated with genetic mutations (usually also referred as genetic resistance), in some cases DR can be developed without chromosomal abnormalities (defined as phenotypic resistance) (Corona and Martinez, 2013). Transcription factors can be involved in both the mechanisms of resistance.

In this paper, we review the impact of transcriptional regulation on drug susceptibility in *M. tuberculosis*.

## Sigma factors and drug susceptibility

Sigma factors are small interchangeable cofactors of RNA polymerase able to confer promoter specificity. The *M. tuberculosis* genome encodes 13 sigma factors and it is the obligate pathogen with the higher amount of sigma factor genes per megabase (Rodrigue et al., 2006). Among these sigma factors, only σ^A^ is essential. The others are dispensable and are activated in response to specific environmental signals. Following their activation, sigma factors switch RNA polymerase holoenzyme promoter-specificity, resulting in a quick change of the bacterial transcriptome leading to the adaptation to the new environment experienced by the bacterium. Mycobacterial sigma factors have been shown to be involved in the response to different conditions endangering the bacterial cells as oxidative stress, alkaline stress, surface stress, low pH, hypoxia, nutrient depletion and heat shock. Drug treatment has been shown to induce a strong stress response in bacteria and the bactericidal activity of some drugs has been primary linked to the stress they induce on the bacteria (Kohanski et al., 2007). Since sigma factors have a primary role in stress response, it is simple to imagine their implication in the establishment of the baseline resistance to drugs.

At least five *M. tuberculosis* sigma factors have been shown to be involved in the basal level of resistance to drugs (**Table 1**). σ^F^ (*i*) is induced upon treatment with ethambutol (EMB), rifampin (RIF), streptomycin (STR), and cycloserine (CS) (Michele et al., 1999), while a *sigF* null mutant in CDC1551 is more resistant to RIF (Chen et al., 2000). However, this phenotype was not confirmed in the H37Rv genetic background, suggesting a different role of σ^F^ in different mycobacterial strains (Hartkoorn et al., 2010).

**Table 1:** Principal transcription factors (TF) in *M. tuberculosis* with established mechanisms of action involved in drug resistance (DR) (transcription factors known for their homology with other mycobacteria are described in the text but not reported in this table).

TF	EXPERIMENTAL CONDITION TESTED	TARGET	EFFECT DURING DRUG-RELATED STRESS	REFERENCES
**SigB**	Deletion		Increased sensitivity to INH and EMB	(Provvedi et al., 2009)
**SigE**	Deletion		Increased sensitivity to VAN, RIF, STR, gentamicin, INH, PZA and EMB	(Pisu et al., 2017)
**SigE**	Constitutive expression		Sensitive to PZA	(Thiede et al., 2022)
**SigF**	Deletion		Increased resistance to RIF*	(Hartkoorn et al., 2010)
**SigH**	Deletion	*Rv2466c/dsbA*	Increased resistance to TP053	(Manganelli et al., 2002; Rosado et al., 2017)
**SigI**	Deletion	*katG*	Increased resistance to INH	(Lee et al., 2012)
**WhiB3**	(Observed) overexpression		Increased tolerance to RIF, MFX, MTX and AMK	(Rodriguez et al., 2014)
**WhiB3**	(Observed) overexpression	*egt* operon (*Rv3700c-Rv3704c*)	Increased sensitivity to RIF, INH, BDQ and CFZ	(Saini et al., 2016; Mavi et al., 2020)
**WhiB4**		β-lactamase	Tolerance to ampicillin	(Mishra et al., 2017)
**WhiB7**	(Mutated) over-expression	*eis, erm37* and *tap*	Increased resistance to STR, LZD, KAN, AMK	(Reeve et al., 2013; Köser et al., 2013; Vargas et al., 2021)
**WhiB7**	Inactivation	*eis, erm37* and *tap*	Hypersusceptibility to macrolides and clarithromycin**	(Li et al., 2022)
**Rv0023**	(Observed) overexpression	*ndh*	Tolerance to INH and ETO	(Gupta et al., 2020)
**Rv0273c**		*inhA*	Increased sensitivity to INH	(Zhu et al., 2018)
**Rv0324**	Deletion		Hypersensitivity to BDQ	(Peterson et al., 2016)
**Rv0678**	(Mutated) Inactivation	*mmpS5-mmpL5* operon	Resistance to BDQ and CFZ	(Kadura et al., 2020)
**Rv0880**	Deletion		Hypersensitivity to BDQ	(Peterson et al., 2016)
**Rv1152**	(Observed) overexpression		Resistance to VAN	(Zeng et al., 2016; Deng et al., 2022)
**Rv3082c**	(Mutated) overexpression	*mymA* operon (*Rv3083 to Rv3089*)	Resistance to ETO and thiooxadiazole 3	(Grant et al., 2016)

*Only in CDC1551 genetic background; **lineage-specific mutation (L1).

**Figure 1:**
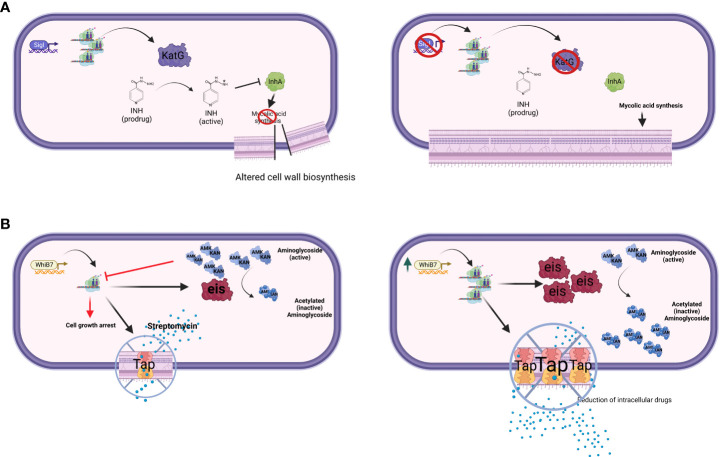
The figure schematizes two major drug-resistance mechanisms mediated by transcriptional factors. **(A)** Transcriptional factor down-regulation. The ECF sigma factor SigI controls the transcription of *katG; m*utant strains lacking SigI* *are more resistant to* *INH (Lee et al., 2012). **(B)** Transcriptional factor over-expression. WhiB7 regulon contains key genes involved in DR, such as *rv2416c* (*eis*) linked to low-level KAN and AMK resistance, and *rv1258c* (*tap*) whose overexpression is linked to low-level STR resistance (Reeves et al., 2013). Point mutations in *tap, *V219A and S292L, have been associated with resistance to PZA, INH, and STR (Liu et al., 2019). Created with BioRender.com.

The extracytoplasmatic function (ECF) sigma factor σ^I^ (*ii*) directly regulates the expression of the structural gene of KatG, an enzyme required for the activation of isoniazide (INH) (**Figure 1A**). Consequently, a *sigI* null mutant was more resistant to this drug both in axenic culture and during mice infection, but surprisingly was not attenuated as predictable for a strain expressing lower levels of KatG (Lee et al., 2012).

The ECF sigma factor σ^H^ (*iii*) is involved in oxidative stress response. One of the genes most induced by σ^H^ in response to oxidative stress is *rv2466c* (Manganelli et al., 2002), encoding for the mycothiol-dependent reductase DsbA (Rosado et al., 2017). This gene is required for the activation of TP053, a promising thienopyrimidine derivative prodrug, able to kill replicating and non-replicating *M. tuberculosis* (Albesa-Jové et al., 2015). Consistent with these data, a *sigH* null mutant was shown to be resistant to TP053 (Rosado et al., 2017). Since *sigH* is induced upon oxidative stress, it is possible to assume that the activation of TP053 activation increases after intracellular bacteria are exposed to reactive oxygen intermediates.

Both σ^B^ (*iv*), a member of the primary-like sigma factors, and the ECF sigma factor σ^E^ (*v*) have been shown to be induced upon exposure to vancomycin (VAN) (Provvedi et al., 2009), and involved in the baseline resistance to several antitubercular drugs. In particular, Pisu and colleagues (Pisu et al., 2017) showed that a *sigE* null mutant was more sensitive to VAN, RIF, STR, gentamicin, INH, and EMB, while a *sigB* null mutant was more sensitive to INH and EMB. Moreover, Yang and colleagues reported that deletion of *sigB* causes increased sensitivity to *para*-aminosalicylic acid and sulfamethoxazole (Yang et al., 2017). Interestingly, Pisu and colleagues showed that σ^B^ and σ^E^ are also essential for the development of persistent bacteria able to survive the bactericidal activity of very high concentrations of VAN (*sigE* mutant), STR and INH (both mutants). These data support the hypothesis that σ^E^ represents a bistable switch involved in persistence development (Balázsi et al., 2008; Sureka et al., 2008; Manganelli and Provvedi, 2010; Tiwari et al., 2010; Zorzan et al., 2021).

Finally, a recent paper demonstrated that σ^E^-mediated activation of surface stress response is essential for pyrazinamide (PZA) susceptibility. PZA is only active when bacteria are exposed to low pH, while its derivative pyrazinoic acid is active also at circumneutral pH. However, a mutant overexpressing *sigE* due to the deletion of the gene encoding its anti-sigma factor RseA (Boldrin et al., 2019) was equally sensitive to PZA at both low pH and circumneutral pH, while a *sigE* null mutant was resistant to both PZA and pyrazinoic acid demonstrating that the activation of the σ^E^ regulon is essential for PZA susceptibility (Thiede et al., 2022). Interestingly, both meropenem and CS, showed synergistic activity with PZA due to their activation of σ^E^-mediated surface stress response (Thiede et al., 2022). Since it is well known that σ^E^ is activated at low pH (Bush, 2018), these data strongly suggest that the role of low pH in PZA susceptibility is the activation of the σ^E^ response.

## WhiB family (WhiB1-7)

*M. tuberculosis* genome encodes seven proteins belonging to the WhiB superfamily. Proteins belonging to this family are small transcriptional regulators and are exclusively present in Actinobacteria. They are characterized by four cysteine residues that bind a [4Fe–4S] cluster and by a five residues (G[V/I]WGG) motif, while their putative role is that to sense O_2_ and nitric oxide (Bansal et al., 2017). Two of them (WhiB1 and WhiB2) are essential for growth, while others were shown to be important for several aspects of mycobacterial physiology as redox homeostasis, DR, dormancy and reactivation (Bansal et al., 2017).

WhiB transcriptional factors are involved in stress response and their genes are often upregulated in multidrug resistant (MDR) clinical isolates. Many *whiB* genes showed altered expression during drug treatment: *whiB6* is downregulated by CS, *whiB2* is upregulated by CS, EMB and INH, *whiB7* is upregulated by macrolides, fluoroquinolones and aminoglycosides (Morris et al., 2005; Geiman et al., 2006; Burian et al., 2012; Chatterjee et al., 2013).

Within the WhiB family, a key player in DR is WhiB7, a transcription factor inducing stress response and promoting low levels of resistance to several antibacterial drugs including macrolides, tetracyclines, and some aminoglycosides (Morris et al., 2005; Burian et al., 2013; Reeves et al., 2013). Recently, an elegant experiment based on CRISPR interference identified *whiB7* as the unique gene responsible for increased susceptibility to ribosome-targeting drugs STR and linezolid (LZD) (Li et al., 2022). Among the genes relevant for DR to antibiotics inhibiting translation (i.e. macrolides), *rv2416c* (*eis*) encodes an aminoglycoside acetyltransferase which inactivate the drugs, whereas 23S rRNA methylation by Rv1988 (Erm37) confers resistance to macrolides. Another gene found to be associated with DR to different drugs including STR is *rv1258c* (*tap*), which encodes for an efflux pump. All these DR genes are part of the WhiB7 regulon (**Figure 1B**). Of particular relevance is the link between mutations affecting the expression of *whiB7* observed by Reeves and colleagues leading to enhanced expression of *rv2416c* (*eis*) (low-level KAN and amikacin resistance) and of *rv1258c* (*tap*) (low-level STR resistance) (Reeves et al., 2013). Interestingly, loss-of-function mutations in the arginine biosynthesis pathway were found to up-regulate the expression of *whiB7*, and *eis* genes, thus conferring tolerance to KAN (Schrader et al., 2021). These mutations mapping in the *rv2747* (*argA*) and *rv1655* (*argD*) genes were associated with increased survival during RIF exposure, as well as with minimum inhibitory concentration (MIC) increase to clarithromycin, again related to the up-regulation of *whiB7*. Modern Beijing isolates and one phylogenetically intermediate Beijing isolate harbored a loss-of-function mutation in *tap*; thus *whiB7* mutations would not translate into low-level STR resistance, but would lead to low-level KAN and AMK resistance only, despite this epistatic interaction has not been proven in clinical isolates yet (Köser et al., 2013; Vargas et al., 2021). Interestingly, a lineage-specific mutation present in the L1 Indo-Oceanic clade inactivates WhiB7, thus making strains belonging to this phylogenetic branch hypersusceptible to macrolides and clarithromycin (Li et al., 2022).

WhiB7 is involved also in physiological stress responses and virulence (Buriánková et al., 2004; Geiman et al., 2006; Zaunbrecher et al., 2009; Homolka et al., 2010; Adams et al., 2011; Kim et al., 2012; Larsson et al., 2012; Ramón-García et al., 2012). Interestingly, the role of WhiB7 in providing intrinsic DR is dependent on its interactions with the principal sigma factor σ^A^, and mutations in either *whiB7* or *sigA* preventing their interaction, have been found to cause multidrug susceptibility (Burian et al., 2013; Lilic et al., 2021). In *M. smegmatis*, WhiB7 was found to positively regulate *ms3140*, a gene homolog of *rv1473* and encoding for an ABC efflux pump involved in macrolide transport (Duan et al., 2019). A similar central role for WhiB7 in DR is found in Actinobacteria, including other mycobacterial opportunistic pathogens such as *Mycobacterium abscessus* (Ramón-García et al., 2013; Hurst-Hess et al., 2017; Pryjma et al., 2017).

The role of other WhiB family members is more nuanced. WhiB4 regulates β-lactamase expression, thus inducing antibiotic tolerance in *M. tuberculosis* (Mishra et al., 2017). WhiB2 is part of a regulatory loop involving *rv1830* (*mcdR*) ultimately fine-tuning mycobacterial cell division and adaptation to stress response, including increased mutation rates during antibiotic challenge (Zhou et al., 2022). Recent genome-wide association analysis (GWAS) approaches have also identified novel associations between mutations in the *whiB6* region and aminoglycosides resistance (Farhat et al., 2019). Mutations in *whiB2* and *whiB6* have been observed within patient microevolution during antimycobacterial treatment, however their role in DR was not fully elucidated (Liu et al., 2015; Xu et al., 2018). WhiB3 was the only transcriptional regulator whose structural gene was induced in a model of adaptation to growth with lipids as the sole carbon source (Rodríguez et al., 2014). In this lipid environment, WhiB3 drove increased drug tolerance to RIF, moxifloxacin (MFX), metronidazole (MTZ), and AMK. Interestingly, similar to WhiB7, WhiB3 was found to interact with σ^A^ (Burian et al., 2013). Several antimycobacterial compounds can produce an oxidative burst as part of their antimicrobial mechanism (Gurumurthy et al., 2013; Piccaro et al., 2014; Shetty and Dick, 2018). Therefore, transcriptional factors involved in redox homeostasis are relevant in maintaining a reducing microenvironment to avoid DNA damage and macromolecules (i.e. protein and small RNA) misfolding. WhiB3 has been described to negatively regulate the *egt* operon (*rv3700c-rv3704c*) encoding for ergothioneine, which together with mycothiol, plays an important role in maintaining the oxidoreduction balance within the bacterial cell (Saini et al., 2016). Both ergothioneine and mycothiol have been proved to be triggered from a wide range of stimuli, from starvation or hypoxia to microenvironmental acidification (i.e. phagolysosome acidification post macrophage infection) (Mavi et al., 2020). Saini and colleagues tested RIF, INH, bedaquiline (BDQ) and clofazimine (CFZ) in *rv3704c* and *rv3701c* deficient strains and observed a MIC reduction for all the tested drugs. Accordingly, resistance and tolerance to antimycobacterial drugs impairing redox homeostasis have been directly linked to the intracellular accumulation of ergothioneine (Saini et al., 2016). On the other side, Xu and colleagues produced several strains with mutations in mycothiol-related enzymes demonstrating that such mutants are resistant to both INH and ethionamide (ETO) (Xu et al., 2011).

## GntR family transcriptional regulators

Transcription factors of the GntR family are widely shared among bacteria, and the firsts members of this family have been described as a gluconate operon repressor in *B. subtilis* (Vindal et al., 2007; Suvorova et al., 2015). GntR members contain a DNA-binding domain with a structural motif helix-turn-helix (HTH) at their N-terminal, conserved among all the family members, and a more variable C-terminal domain that has been used to divide the GntR factors into six subfamilies (Suvorova et al., 2015). Most of the characterized GntR family members are transcriptional repressors, although some exceptions exist. The *M. tuberculosis* genome encodes for a large number of GntR family transcription factors, although their role and regulation are still poorly described (Cole et al., 1998).

Among the techniques used to identify transcription factors potentially relevant for DR, over-expressing libraries for transcriptional regulators under selection on high drug concentrations have been shown to be successful tools. For example, Hu and colleagues (Hu et al., 2015) identified a hypothetical transcription factor encoded by the *ms0535* gene as a potential contributor to INH resistance in *M. smegmatis*. Sequence analysis showed that Ms0535 belongs to the GntR family (FadR sub-family). Ms0535 acts as a transcriptional activator for the expression of its own structural gene and a major facilitator superfamily permease gene *ms0534* in the same operon, thus triggering INH resistance. Interestingly, the two genes are not responsive to INH, although their over-expression increases INH resistance. Both *ms0535* and *ms0534* are absent in *M. tuberculosis*, thus they can contribute to explain the differences in INH resistance between the two species.

In *M. tuberculosis* there are at least eight putative GntR-like proteins: Rv0043c, Rv0165c (Mce1R), Rv0494, Rv0586 (Mce2R), Rv0792c (MoyR), Rv1152, Rv3060c, and Rv3575c (Vindal et al., 2007; Chauhan et al., 2021). Among them, Rv0494 has an ortholog in *M. smegmatis* (Ms2173). *M. smegmatis* mutants for Ms2173 showed altered INH and RIF susceptibility (its over-expression led to increased INH and RIF susceptibility), likely due to the regulatory activity of this transcription factor on membrane-associated transporter genes (Rao et al., 2012).

Rv1152 is involved in the regulation of cell wall permeability (Zeng et al., 2016; Deng et al., 2022). This transcriptional regulator is involved in acid and cell surface stress response and plays an important role in determining VAN resistance by negatively regulating genes responsive to this glycopeptide antibiotic. Indeed, *M. smegmatis* overexpressing *M. tuberculosis* Rv1152 showed an increased resistance to VAN, whereas deleting its homologous gene (*ms5174*) established increased sensitivity that could be restored by complementation with *rv1152* (Zeng et al., 2016).

Deletion of *rv0792c* impaired the ability of *M. tuberculosis* to infect guinea pigs, however no difference was observed in survival during exposure to INH, RIF, or LEV (Chauhan et al., 2021). A role in DR for the remaining GntR family members has yet to be identified, although some of them have been found upregulated in drug resistant isolates. Interestingly, protein levels of Rv0043c were found less abundant in lineage 7 (L7), however the phenotypic outcomes of these findings remain unknown (Yimer et al., 2020).

## Xenobiotic response element (XRE) family transcriptional regulators

XRE response element are among the most widespread regulatory elements in bacteria. They are characterized by a conserved HTH DNA binding domain at their N-terminus and a highly variable C-terminal region. The *M. tuberculosis* genome encodes for seven members of this family: Rv0023, Rv0465c (RamB), Rv0474, Rv1129 (PrpR), Rv2017, Rv2021, and Rv3849 (EspR).

Rv0023 is a transcription factor modulating nearly 900 genes, and its regulon is enriched for NAD reductases (Rustad et al., 2014). Given the link with NADH/NAD^+^ regulation, Gupta and coll. explored the role of Rv0023 in INH and ETO tolerance. The overexpression of Rv0023 conferred increased INH and ETO tolerance in *M. smegmatis* by downregulating the expression of the *ndh* gene, which encodes for a NADH dehydrogenase. This leads to increased NADH cellular concentration and subsequent inhibition of drug-NAD^+^ adducts formation, which are essential for INH activity (Gupta et al., 2020). Furthermore, the study found that Rv0023 is also a negative regulator of *whiB5*.

Rv0465c (RamB), and Rv1129 (PrpR) are involved in the regulation of propionate and acetate metabolisms, respectively. A link between the two metabolic pathways and conditional drug tolerance has been established; however, its biological meaning remains unclear and needs further elucidation (Hicks et al., 2018; Tang et al., 2019; Hicks et al., 2020).

*rv2017* was found to be deleted or disrupted by IS*6110* in several drug resistant isolates; however, the link between this gene and specific drug resistant phenotype has yet to be defined (Klopper et al., 2020; Perdigão et al., 2020; Antoine et al., 2021). Rv3849 (RspR) was predicted to regulate *ponA1*, a gene involved in cell wall synthesis relevant for altered fitness in *M. tuberculosis* during RIF treatment (Farhat et al., 2013; Kieser et al., 2015; Kieser et al., 2015). The remaining XRE transcription regulators in *M. tuberculosis* are uncharacterized for their role in DR.

## MarR family transcriptional regulators

There are at least nine genes in the genome of *M. tuberculosis* annotated as MarR-like proteins. One of the most studied MarR-like family transcriptional regulator is Rv0678. This is a transcriptional repressor of the *mmpS5-mmpL5* operon, which encodes an efflux pump able to transport BDQ and CFZ (Milano et al., 2009; Andries et al., 2014). Mutations in *rv0678* affecting its binding activity to the promoter region of the *mmpS5-mmpL5* operon are relevant markers of BDQ and CFZ resistance in *M. tuberculosis* (Kadura et al., 2020). Peterson and coll. described BDQ tolerance mediated by Rv0880 and Rv0324, regulators belonging to the MarR and ArsR family, respectively (Peterson et al., 2016). Knockout mutant strains for *rv0324* and *rv0880* showed hypersensitivity to BDQ, without affecting the susceptibility to other antimycobacterial drugs with unrelated mechanisms of action. Drug tolerance is mediated by the transcriptional cascades modulated by the two transcription factors rather than being caused by genetic mutations. Interestingly, the Rv0324 regulon correlates with nutrient-limited stress condition, which has important implications since BDQ killing depends upon glycolytic pathways (Mackenzie et al., 2020).

Resistance to a novel pyrido-benzimidazole with potent mycobactericidal activity was found to be mediated by mutations in the gene encoding the MarR-like Rv2887 transcription factor (Warrier et al., 2016; Gao et al., 2017). Mutations in this gene negatively affects the ability of the transcription factor to bind its target DNA sequences, ultimately leading to the upregulation of downstream genes. Among them, *rv0560c* was found to encode for a benzoquinone methyltransferase able to N-methylate and thus inactivate the pyrido-benzimidazole compound (Warrier et al., 2016). Mutations in Rv2887 were also found to abrogate susceptibility to a new imidazopyridine-based drug candidate (Winglee et al., 2015). In this case, DR is mediated by the upregulation of efflux pumps yet to be further identified.

Rv2327 has been hypothesized to participate in INH antibiotic response given its role in the regulation of *fbpA* and *fbpC* (encoding antigen 85 complex A and C, respectively), which are involved in cell wall biosynthesis and over-expressed in response to INH treatment (Nguyen et al., 2005; Romero et al., 2010). However, the putative mechanism (e.g. direct interaction Rv2327-INH or other) has not been further explored. Other members of the family have not been directly linked with DR in *M. tuberculosis* so far.

## TetR family transcriptional regulators

Regulators of the TetR family usually repress transcription binding their target DNA sequence through a conserved HTH motif present at their N-terminus. Target DNA is released following a structural change of the protein caused by the binding of a specific effector molecule to a ligand-binding pocket situated at the C-terminus of the protein (Balhana et al., 2015). Among the pathways under the control of these regulators, drug efflux is probably the most studied. The TetR family regulator Rv3066 was described to repress the transcription of *rv3065 (mmr)*, a small multidrug resistance (SMR) efflux pump (Bolla et al., 2012). Similarly, Rv1219c was reported to regulate the transcription of the ATP-binding cassette (ABC) transporter encoded by *rv1217c-rv1218c* (Kumar et al., 2014). Both the SMR and ABC transporters have been found overexpressed in MDR clinical isolates, however experiments with knockout strains, recently challenged their role in direct transport of relevant anti-TB drugs (Wang et al., 2013; Shahi et al., 2021; Remm et al., 2022). A recent study matching genomic mutations and increased MIC levels to several drugs identified mutations mapping in the low-affinity binding domain of Rv1219c associated with increased MIC for INH, thus proposing that the repression of the *rv1217c-rv1218c* is somehow linked with increased levels of resistance to this first-line drug (Consortium et al., 2021).

Three members of these transcriptional regulators have been directly linked with DR: (*i*) Rv0275 (InbR) is able to directly interact with INH, and its overexpression is associated with increased resistance to this drug, whereas knockout mutants showed increased susceptibility to several anti-TB drugs (Yang et al., 2018). Genes involved in the INH pathway such as *iniABC* were shown to belong to its regulon; (*ii*) *Rv3855* (EthR) is well-known for its regulatory role on *ethA* (*rv3854c*), which encodes a Baeyer-Villager monooxygenase involved in the activation of ETO (Engohang-Ndong et al., 2004). Despite the role of mutations affecting *ethA* in ETO resistance is evident (Ushtanit et al., 2022), mutations in its transcriptional regulator seems relatively rare in clinical isolates (da Silva et al., 2018; Mugumbate et al., 2021); (*iii*) Rv0273c (EtbR) is a transcriptional repressor of the *inhA* gene, which encodes the target of INH. EMB can bind EtbR, increasing its repressing activity on *inhA* transcription, thus increasing susceptibility to INH (Zhu et al., 2018).

Further relationships between TetR family transcriptional regulators and DR levels in *M. tuberculosis* have to be discovered yet. Of note, Rv0302, Rv1816, and Rv3249c are predicted to regulate *mmpL3* and *mmpL11*, two relevant drug target candidates (Domenech et al., 2005; Chou et al., 2015; Delmar et al., 2015).

## Other transcriptional regulators and post-translational modifications affecting transcriptional factors

Among additional transcriptional regulators reported to affect DR in mycobacteria we can mention the members of the AraC/XylS and the SmtB/ArsR families. The AraC/XylS family of transcription factors includes hundreds of positive regulators (Gallegos et al., 1997; Egan, 2002). In *M. tuberculosis* at least nine members of the AraC/XylS family have been described: Rv0023, Rv0465c (RamB), Rv0474, Rv1129 (PrpR), Rv2017, Rv2021, Rv3082c (VirS), and Rv3849 (EspR). Mutations in *rv3082c* (*virS*) were found to mediate resistance to a new putative antimicrobial compound (defined as thiooxadiazole 3) and ETO by affecting the expression of the *mymA* operon, which is responsible for the activation of these molecules (Grant et al., 2016). Other members of the family are involved in several regulatory functions related to carbon metabolism, stress response, and pathogenesis.

At least 12 ArsR family homologs, including Rv0324, Rv2034 and the metal sensors Rv0827c (KmtR), Rv1994c (CmtR), Rv2358 (SmtB), and Rv3744 (NmtR), have been described in *M. tuberculosis.* Beside the already mentioned role of Rv0324 in BDQ tolerance (Peterson et al., 2016), no further links with DR have been found for this family of transcriptional regulators. A study showed the Rv2034, a regulator of the ArsR family, regulates *whiB7* expression, but its role on the WhiB7 regulon in terms of drug tolerance/resistance has not been further explored (Gao et al., 2012).

Additional transcriptional regulators have been reported to affect DR in mycobacteria. For example, the histone-like Lsr2 protein (encoded by the *rv3597c* gene) is involved in several regulatory functions involving cell wall biosynthesis, transport, and responses to antibiotic treatment. Lsr2 represses INH-mediated induction of *iniBAC* and *efpA* (Colangeli et al., 2007). Interestingly, *iniBAC* is also under the control of another transcriptional regulator: IniR (Rv0339c) (Boot et al., 2017). Similarly, another histone-like protein, HupB (Rv2986c, also known as MDP-1), negatively regulates *katG* expression, thus affecting phenotypic tolerance to INH in *M. tuberculosis* (Niki et al., 2012). Another example is Rv1267c (EmbR), which regulates *embABC* transcription, well-known to be relevant for EMB resistance (Sharma et al., 2006). An additional case of interest relates to CFZ. Li and coll. described a link between the transcriptional regulator Rv1453 and CFZ resistance, where the overexpression of the gene was associated with increased MIC to the drug (Li et al., 2021).

Several transcription factors are modified post-translationally, thus adding an additional layer of control that can modify the interaction between bacteria and drugs. Among those previously cited, EmbR is positively regulated by phosphorylation, whereas HupB acetylation and methylation alter INH susceptibility in mycobacteria (Arora et al., 2021). Similarly, PknB phosphorylates the histon-like protein Lsr2, thus reducing its DNA binding affinity (Alqaseer et al., 2019). Rv3701c (part of the *egt* operon), is under the strict regulation of the serine/threonine-protein kinase PknD, and its phosphorylated form fails to catalyzes ergothioneine biosynthesis (Richard-Greenblatt et al., 2015). Therefore, it is plausible that these additional regulatory layers have a role in fine-tuning redox homeostasis, and thus drug tolerance. Moreover, transcription factors are not universally conserved in the *M. tuberculosis complex*, thus this genetic diversity has to be taken into account systematically when studying genotypic features in relationship with phenotypic drug susceptibility (Köser et al., 2012; Chiner-Oms et al., 2018; Chiner-Oms et al., 2019).

## Targeting transcriptional regulation: A new frontier for drug discovery

As DR has become a crucial worldwide problem, new strategies to design innovative generations of antibacterial drugs has been implemented. Targeting transcriptional regulation is one of them. An interesting example is that of EthR. The mycobacterial monoxygenase EthA is the activator of several thiocarbamide-containing drugs, including ETO. Its expression is regulated by EthR, a transcriptional repressor. Synthetic compounds selected to inhibit EthR-DNA interaction have been shown to increase *ethA* expression, boosting bacterial sensitivity to ETO (Willand et al., 2009; Flipo et al., 2012; Nikiforov et al., 2017). Another emerging and promising strategy is targeting riboswitches (Deigan and Ferré-D’Amaré, 2011; Lünse et al., 2014; Dar et al., 2016; Panchal and Brenk, 2021). Metabolite-binding riboswitches are non-coding RNAs that bind specifically to metabolites and regulate downstream gene expression depending on the metabolite concentration. These sequences are composed of two domains: an aptamer that binds the metabolites/ligands, and a response platform that allows the expression or repression of downstream genes. The binding of ligand leads either to transcriptional and/or translational termination of downstream gene expression (switch-OFF) or to the expression of downstream genes (switch-ON) (Bastet et al., 2018; Yadav et al., 2020). Riboswitches occur almost exclusively in prokaryotes and are involved in the metabolism of essential amino acids and metabolites. Although not yet thoroughly studied, some riboswitches are present in pathogenic bacteria, including *M. tuberculosis*, and play an important role in controlling essential genes. Indeed, many of the classes of riboswitches are fundamental in controlling the expression of genes involved in virulence (Blount and Breaker, 2006; Lünse et al., 2014). Being involved in the expression of essential genes and absent in eukaryotic genomes, riboswitches have become interesting targets for the design of innovative antibacterial strategies. The general idea is to target the pocket recognizing the metabolite to induce a transition of the riboswitch from the ON to the OFF configuration turning off the regulated gene. Riboswitches-based antibacterial drugs could be broad-spectrum, in the case of riboswitches present in an extended range of pathogenic species, or narrow spectrum in the case of species-specific riboswitches (Blount and Breaker, 2006; Panchal and Brenk, 2021). Using this rational some antibacterial compounds targeting riboswitches have already been identified (recently reviewed in [Panchal and Brenk, 2021)]. *M. tuberculosis* is predicted to have at least 16 riboswitches from the Rfam database belonging to different classes (Kalvari et al., 2018). However, only few riboswitches have been validated in this species (Schwenk and Arnvig, 2018) as the cyclic-di-AMP sensing riboswitch regulating *rpfA* (Arnvig and Young, 2012; Nelson et al., 2013), the riboswitch regulating *rpfB* (Schwenk et al., 2018), and the cobalamin-dependent riboswitch responsible for the downregulation of *metE* in the presence of cobalamin (Warner et al., 2007). The latter case is particularly interesting to show the potential importance of riboswitches as drug targets. *M. tuberculosis* has two methionine synthases: MetE, whose expression is repressed by cobalamin and MetH, which requires cobalamin for its functionality. In the clinical isolate CDC1551 MetH is not functional due to a mutation in its structural gene, so MetE is the only methionine synthase in this strain. Warner and coll. (Warner et al., 2007) showed that indeed this strain is unable to grow in the presence of cobalamin due to the repression of *metE* expression. Mutants resistant to cobalamin showed mutations at the level of the riboswitch confirming the role of this regulatory element in *metE* regulation.

## Conclusions

Drug exposure represents a stress for the bacterium, which reacts by changing its transcriptional profile thus activating different stress regulons responsible of setting its own basal level of susceptibility to a specific drug. Sometimes, mutations in transcriptional regulators can structurally modify the bacterial transcriptional profile resulting in a constitutive change in the susceptibility to a given drug. Moreover, as outlined above, post-transcriptional regulation can further modify transcriptional regulation.

Understanding the role of transcription factors in DR pathways is critical not only to improve our knowledge in resistance mechanisms or to detect new genetic markers of resistance. Unravelling the transcriptomic networks would indeed open to new therapeutic opportunities. As an example, it has been recently reported how DR in *M. tuberculosis* could give rise to collateral sensitivity to β-lactam drugs, where basically anti-TB drugs induces the expression of the gene encoding the transcriptional repressor BlaI and its downstream genes *atpH*, and *sigC*, which ultimately inhibits intrinsic β-lactam resistance (Trigos et al., 2021). Finally, small non-coding RNAs and riboswitches have been described to modulate antibiotic tolerance, and resistance in several bacteria (Dar et al., 2016; Dersch et al., 2017; Zhang et al., 2020). The role (if any) in DR of transcriptional and/or post-transcriptional regulation mediated by these molecules in *M. tuberculosis* has not been elucidated yet and requires further investigation.

## Author contributions

PM: Writing - original draft, conceptualization, investigation. RS: Writing - original draft, visualization, investigation. SG; Writing - review & editing, investigation. RP: Writing - review & editing. DMC: Writing - review & editing. RM: Writing - original draft, conceptualization, investigation, supervision. All authors contributed to the article and approved the submitted version.

## Funding

RM laboratory is founded from the Innovative Medicines Initiative 2 Joint Undertaking (JU) under grant agreement no 853989. RS was supported by the Italian Ministry of Health “Ricerca Finalizzata 2016” under grant agreement GR-2016-02364014 to PM.

## Conflict of interest

The authors declare that the research was conducted in the absence of any commercial or financial relationships that could be construed as a potential conflict of interest.

## Publisher’s note

All claims expressed in this article are solely those of the authors and do not necessarily represent those of their affiliated organizations, or those of the publisher, the editors and the reviewers. Any product that may be evaluated in this article, or claim that may be made by its manufacturer, is not guaranteed or endorsed by the publisher.
